# Next-Generation Sequencing Identifies Pathogenic Variants in *HGF*, *POU3F4*, *TECTA*, and *MYO7A* in Consanguineous Pakistani Deaf Families

**DOI:** 10.1155/2021/5528434

**Published:** 2021-04-22

**Authors:** Xueshuang Mei, Yaqi Zhou, Muhammad Amjad, Weiqiang Yang, Rufei Zhu, Muhammad Asif, Hafiz Muhammad Jafar Hussain, Tao Yang, Furhan Iqbal, Hongyi Hu

**Affiliations:** ^1^Department of Otorhinolaryngology, Peking University Shenzhen Hospital, Shenzhen, China; ^2^Department of Otorhinolaryngology, Peking University Shenzhen Hospital, Shenzhen Peking University-The Hong Kong University of Science and Technology Medical Center, Shenzhen, China; ^3^Institute of Pure and Applied Biology, Bahauddin Zakariya University, Multan, Pakistan; ^4^Department of Nephrology, Institute of Nephrology, Ruijin Hospital, Shanghai Jiao Tong University School of Medicine, Shanghai, China; ^5^Department of Otorhinolaryngology-Head and Neck Surgery, Ninth People's Hospital, Shanghai Jiao Tong University School of Medicine, Shanghai, China; ^6^Ear Institute, Shanghai Jiao Tong University School of Medicine, Shanghai, China; ^7^Shanghai Key Laboratory of Translational Medicine on Ear and Nose Diseases, Shanghai, China

## Abstract

**Background:**

Approximately 70% of congenital deafness is attributable to genetic causes. Incidence of congenital deafness is known to be higher in families with consanguineous marriage. In this study, we investigated the genetic causes in three consanguineous Pakistani families segregating with prelingual, severe-to-profound deafness.

**Results:**

Through targeted next-generation sequencing of 414 genes known to be associated with deafness, homozygous variants c.536del (p. Leu180Serfs∗20) in *TECTA*, c.3719 G>A (p. Arg1240Gln) in *MYO7A*, and c.482+1986_1988del in *HGF* were identified as the pathogenic causes of enrolled families. Interestingly, in one large consanguineous family, an additional c.706G>A (p. Glu236Lys) variant in the X-linked *POU3F4* gene was also identified in multiple affected family members causing deafness. Genotype-phenotype cosegregation was confirmed in all participating family members by Sanger sequencing.

**Conclusions:**

Our results showed that the genetic causes of deafness are highly heterogeneous. Even within a single family, the affected members with apparently indistinguishable clinical phenotypes may have different pathogenic variants.

## 1. Background

Congenital hearing loss affects 1‰-2‰ of newborns worldwide. Among them, approximately 70% of deafness is attributable to genetic causes [1]. The genetics of hearing loss is extremely heterogeneous as, to date, more than 120 genes are reported to cause nonsyndromic hearing loss (Hereditary Hearing Loss Homepage; https://hereditaryhearingloss.org). Similarly, numerous genes are known to cause syndromic hearing loss [2–10]. In recent years, next-generation sequencing (NGS) has been increasingly implemented in the genetic diagnosis of heterogeneous diseases including hearing loss, providing a high-throughput, efficient approach to target hundreds of causative genes or even the whole exome for the identification of pathogenic variants [11, 12]. Due to the extremely high heterogeneity of genetic hearing loss, however, determining the pathogenicity of the candidate variants can be challenging in many cases. For example, rare, benign variants are not always distinguishable from the true pathogenic variants following general guidelines for sequencing data interpretation [13, 14]. Reporting of rare variants with phenotypic cosegregation in large families, therefore, will provide valuable references for genetic diagnosis of deafness in isolated cases [14].

The deafness-associated genes play diverse roles in the development, function, and maintenance of the inner ear. Variants in these genes correspondently lead to variable auditory phenotypes in regard to onset, severity, progression, audiogram profile, and accompanying syndromic features [1]. In some cases, different variants in the same gene, such as *MYO7A*, may result in distinct phenotypes in nonsyndromic deafness *DFNA11* and *DFNB2* [15, 16] or syndromic deafness *USH1B* [17]. Documentation and analysis of the genotype-phenotype correlation covering a broad range of novel or previously less characterized variants are therefore necessary to facilitate genetic diagnosis of hearing loss.

Approximately 80% of genetic deafness are inherited in an autosomal recessive manner [18]. In many cases, they are closely related to consanguineous marriage, which is common in regions of the Middle East, South Asia, South America, and Africa [19, 20]. Studies in Iranian and Palestinian populations, for example, showed that, respectively, 65% and 58% of deaf children were born to parents having consanguineous marriage [21, 22]. A number of deafness-associated genes are discovered by linkage analyses based on homozygosity mapping of large consanguineous deaf families [23, 24].

In this study, we performed targeted NGS in three Pakistani consanguineous families and identified novel variants in *TECTA* and *POU3F4* and previously reported variants in *MYO7A* and *HGF* as the pathogenic causes for prelingual, severe-to-profound deafness. Interestingly, in a large, multigenerational consanguineous family, we identified two separated variants in *HGF* and *POU3F4*, illustrating the complex genetic heterogeneity of deafness.

## 2. Materials and Methods

### 2.1. Subjects and Clinical Evaluations

**Figure 1:**
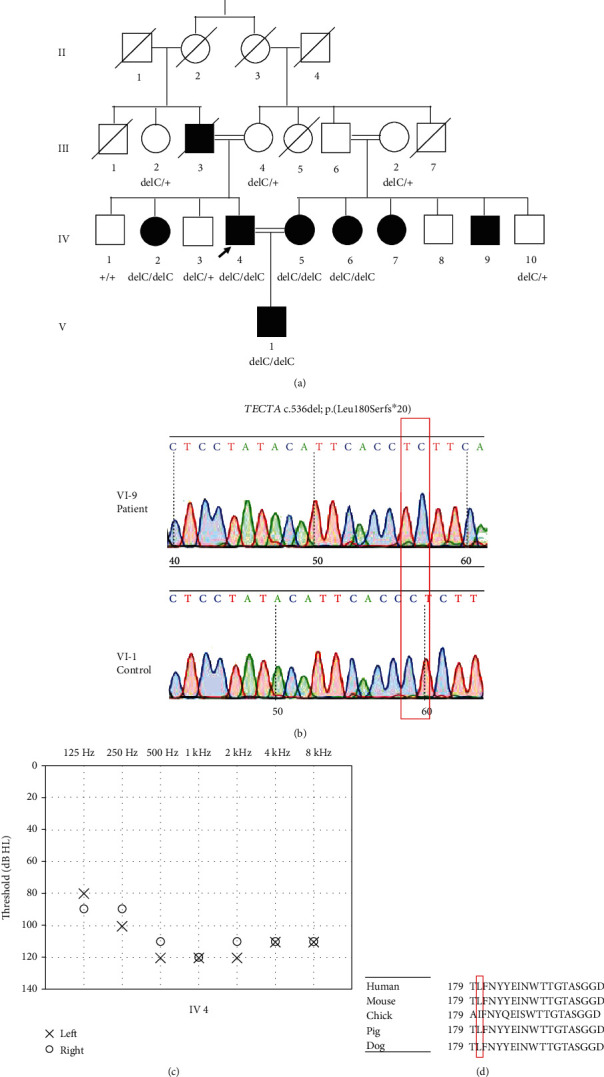
Genetic and phenotypic characterization of Family PK-DD-KA-01. (a) Pedigree and genotype showing the c.536del (p. Leu180Serfs∗20) variant in *TECTA*. (b) Sanger sequencing of the c.536del (p. Leu180Serfs∗20) variant in affected and unaffected family members. (c) Pure tone audiometry showing the bilateral profound hearing loss in affected family member IV-4. (d) Multiple sequence alignment showing the conservation of the L180 residue in different species.

**Figure 2:**
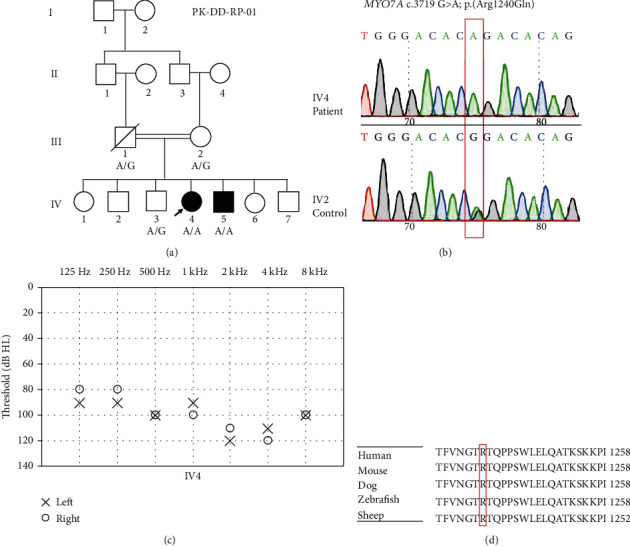
Genetic and phenotypic characterization of Family PK-DD-RP-01. (a) Pedigree and genotype showing the c.3719 G>A (p. Arg1240Gln) variant in *MYO7A*. (b) Sanger sequencing of the c.3719 G>A (p. Arg1240Gln) variant in affected and unaffected family members. (c) Pure tone audiometry showing the bilateral profound hearing loss in affected family member IV-4. (d) Multiple sequence alignment showing the conservation of the R1240 residue in different species.

**Figure 3:**
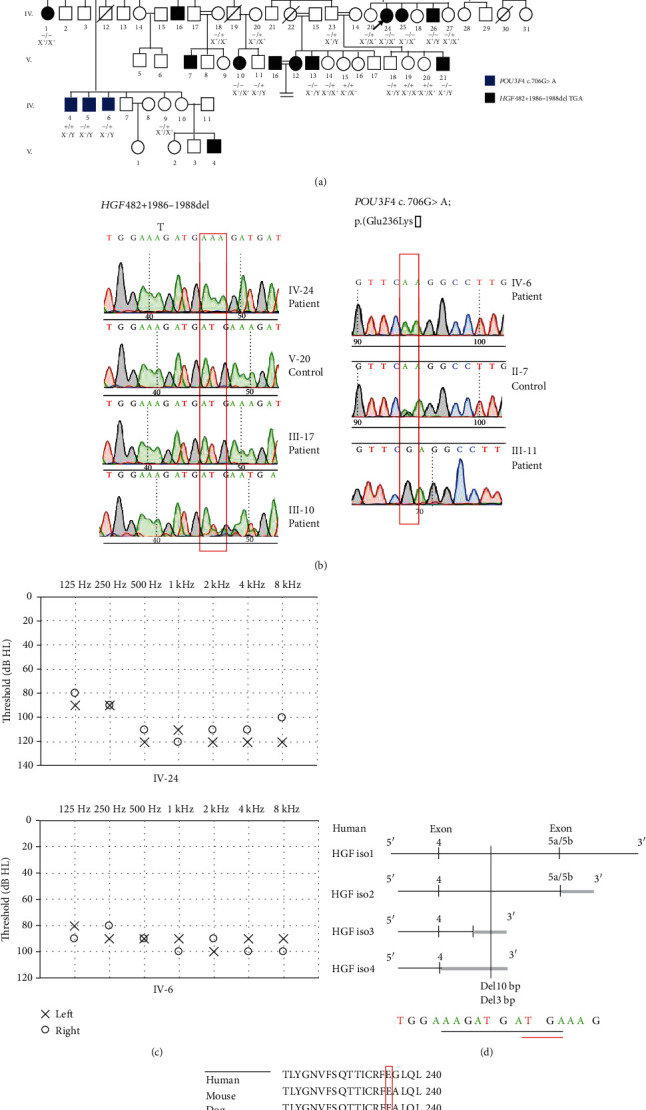
Genetic and phenotypic characterization of Family PK-DB-OKA-01. (a) Pedigree and genotypes showing the 482+1986-1988del variant in *HGF* (marked as -) and the c.706G>A (p. Glu236Lys) variant in *POU3F4* (marked as X^−^). (b) Sanger sequencing of the 482+1986-1988del and c.706G>A (p. Glu236Lys) variants. (c). Pure tone audiometry showing the bilateral profound hearing loss in affected family members IV-24 and IV-6. (d) Position of the 3 bp deletion in human *HGF* and the 10 bp deletion in mouse *Hgf* that both lead to profound hearing loss. (e) Multiple sequence alignment showing the conservation of the E236 residue in different species.

Three consanguineously married families (PK-DD-KA-01 (Figure 1), PK-DD-RP-01 (Figure 2), and PK-DB-OKA-01 (Figure 3)) were enrolled from three districts (Muzafargarh, Rajanpur, and Okara, respectively) in Punjab (Pakistan) with multiple individuals suffering from deafness. Written informed consents were obtained from all participants and/or their parents before their enrollment in this study. Family members affected with deafness were examined in the ear, nose, and throat (ENT) wards of their respective District Head Quarter Hospital by medical specialists. Clinical evaluations included complete medical history interview, comprehensive physical examination, and pure tone audiometry (PTA). Otoscopic examination was performed to exclude hearing loss due to infections, trauma, or other environmental factors. The vestibular function was evaluated by medical history inquiry and behavioral testing. This study was approved by the Research Ethics Committee of the Peking University Shenzhen Hospital.

### 2.2. Genetic Analysis

Genomic DNA was extracted from peripheral blood samples of the enrolled subjects by using the QIAamp DNA Blood Mini Kit (QIAGEN, Shanghai). Targeted NGS was performed in the proband from each family (arrows in Figures 1(a), 2(a), and 3(a)) plus individual IV-5 from Family PK-DB-OKA-01. The customized capture panel (MyGenostics, Beijing) targeted the exonic region of 414 known deafness-associated genes; in addition to the exonic region, the panel also included all intronic, intergenic, and noncoding regions containing variants that reported in HGMD (147 genes, 751 variants) (Supplementary Table S1). Candidate pathogenic variants were defined as nonsynonymous (including nonsense, missense, splice-site, and indels) variants with minor allele frequencies (MAFs) less than 0.005 in public databases including 1000 Genomes, dbSNP, and GnomAD. Potential pathogenic effect of the candidate variants was evaluated by *in silico* tools MutationTaster, PROVEAN, SIFT, and PolyPhen-2. Cosegregation of the deafness phenotype and the pathogenic variants was confirmed in participating family members by PCR amplification and Sanger sequencing; the results were shown in each pedigree map. Pathogenicity of the variants was classified following the guidelines of ACMG 2015 [13].

## 3. Results

### 3.1. Clinical Characterization

There are at least seven subjects in family PK-DD-KA-01 (Figure 1(a)), 2 in PK-DD-RP-01 (Figure 2(a)), and 22 in PK-DB-OKA-01 (Figure 3(a)) that were suffering from bilateral, prelingual, severe-to-profound sensorineural hearing loss (Figures 1(c), 2(c), and 3(c)). All affected subjects in Families PK-DD-KA-01 and PK-DD-RP-01 and half (11/22) of the affected subjects in Family PK-DB-OKA-01 were born to parents with consanguineous marriage. No ear malformation, vestibular dysfunction, developmental abnormality, or syndromic symptom were identified in enrolled subjects.

### 3.2. Identification of the Pathogenic Variants in Probands

Targeted NGS of 414 known deafness genes was performed on probands of the three families. Homozygous variants c.536del (p. Leu180Serfs∗20) in *TECTA* (NM_005422.4), c.3719 G>A (p. Arg1240Gln) in *MYO7A* (NM_001127180.2), and c.482+1986_1988del in *HGF* (NM_000601.6) were identified as the candidate pathogenic variants in probands IV-4 of Family PK-DD-KA-01 (Figure 1(a)), IV-4 of Family PK-DD-RP-01 (Figure 2(a)), and IV-24 of Family PK-DB-OKA-01 (Figure 3(a)), respectively. All candidate variants have minor allele frequencies lower than 0.0001 in the public database gnomAD and are categorized as likely pathogenic based on ACMG guidelines (Table 1).

### 3.3. Identification of a Second Pathogenic Variant in Family PK-DB-OKA-01

Sanger sequencing confirmed that homozygous variants c.536del (p. Leu180Serfs∗20) in *TECTA* and c.3719 G>A (p. Arg1240Gln) in *MYO7A* segregated with the deafness in all participating members in Families PK-DD-KA-01 (Figure 1(b)) and PK-DD-RP-01 (Figure 2(b)), respectively. In Family PK-DB-OKA-01, however, five deaf members (IV-4, IV-5, IV-6, III-10, and III-17, marked blue in (Figure 3(a))) were either heterozygous or wild type for variant c.482+1986_1988del in *HGF* (Figure 3(b)). A second round of targeted NGS on subject IV-5 in this family identified a hemizygous c.706G>A (p. Glu236Lys) variant in the X-chromosome gene *POU3F4* (NM_000307.5), a causative gene for X-linked nonsyndromic deafness *DFNX2*. The c.706G>A (p. Glu236Lys) variant segregated with the deafness in the five aforementioned male family members, but it was not detected in any other family members (Figures 3(a) and 3(b)). This novel variant is not seen in the gnomAD databases and has not been previously reported in association with hearing loss. It substitutes an evolutionarily conserved, acidic residue glutamic acid to an alkaline residue lysine at position 236 (Figure 3(e)) and is predicted to be deleterious or probably damaging by *in silico* tools MutationTaster, PROVEAN, SIFT, and PolyPhen-2 (Table 1).

## 4. Discussion

Hearing loss is one of the major disabilities worldwide, which is often induced by loss of sensory hair cells [25–29] and spiral ganglion neurons [30–34] in the inner ear cochlea. Hearing loss could be caused by genetic factors, aging, chronic cochlear infections, infectious diseases, ototoxic drugs, and noise exposure [35–42], and genetic factors account for around 70% of the hearing loss. In the present study, we are reporting the genetic causes of the prelingual, severe-to-profound deafness in three consanguineous Pakistani families through targeted NGS approach. Variants c.536del (p. Leu180Serfs∗20) in *TECTA* and c.706G>A (p. Glu236Lys) in *POU3F4* are novel, while c.3719 G>A (p. Arg1240Gln) in *MYO7A* and c.482+1986_1988del in *HGF* were previously reported in only very limited cases associated with deafness [43, 44]. In support of their pathogenicity, all four variants segregated with multiple affected and unaffected family members consistent with the autosomal recessive or X-linked inheritance modes. Considering that homozygosity of rare variants is quite rare in the general population, the data generated during the present study will provide valuable genetic evidence regarding the genetic basis of deafness.

**Figure 4:**
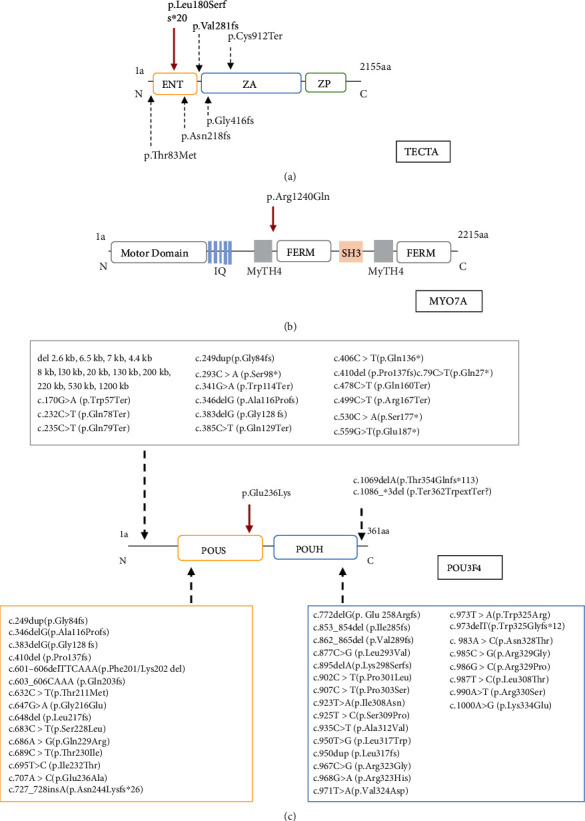
Domain structure and variant position of TECTA, MYO7A, and POU3F4 proteins. (a) *α*-Tectorin consists of entactin domain (ENT), zonadhesin (ZA) domain, and C-terminal zona pellucida (ZP) domain. Previously reported autosomal recessive variants in *TECTA* are presented, with the c.536del (p. Leu180Serfs∗20) variant highlighted by the red arrow. (b) Myosin VIIA consists of a motor domain, five IQ motif repeats, two large repeats of MyTH4 and FERM domains, and an SH3 domain. The c.3719 G>A (p. Arg1240Gln) variant is highlighted by the red arrow. (c) POU3F4 consists of a POU-specific domain (POUS) and a POU homeodomain (POUH). Previously reported *POU3F4* variants are presented, with the c.706G>A (p. Glu236Lys) variant highlighted by the red arrow.

The phenotypes of the three families in this study are all characterized as prelingual, severe-to-profound sensorineural hearing loss. Consistently, a similar type of hearing loss has also been associated with many variants in *TECTA*, *MYO7A*, *HGF*, and *POU3F4* in previous reports. *TECTA* encodes *α*-tectorin, which is one of the main components to comprise the tectorial membrane in the cochlea [45]. Unlike dominant *TECTA* variants, which are associated with milder hearing loss DFNA8/12 (MIM 601543), the recessive *TECTA* variants often result in prelingual, severe-to-profound hearing loss (DFNB21, MIM 603629) [46–50]. Like several other recessive, truncating variants in *TECTA*, the c.536del (p. Leu180Serfs∗20) variant identified in this study causes a frameshift; this will result either in an abortive protein truncated in exon 4 or in no protein at all due to nonsense-mediated mRNA decay [51] and likely results in loss of function (Figure 4(a)). *MYO7A* is extensively expressed in the hair cells and plays an important role in maintaining stereocilia differentiation and morphology [52, 53]. The c.3719 G>A (p. Arg1240Gln) variant in *MYO7A* locates in the highly conserved first MyTH4 domain (Figure 4(b)). It has been previously reported to be associated with Usher syndrome 1B (MIM 276900), characterized by congenital, severe-to-profound hearing loss, and late-onset retinitis pigmentosa before or after puberty [44, 54]. Since the two affected children with the c.3719 G>A (p. Arg1240Gln) homozygous variant in our study were 7 and 9 years old without apparent visual abnormality, the potential visual dysfunction remains to be determined at older age. *HGF* encodes a hepatocyte growth factor and is expressed in the stria vascularis of the mouse inner ear. The c.482+1986_1988del variant identified in this study has been previously reported to cause prelingual, profound deafness (DFNB39, MIM 608265) [43]. A *Hgf* 10 bp deletion in homozygous mutant mice, which fully encompasses the 3 bp deletion in humans, also displayed profound hearing loss at 4 weeks age (Figure 3(d)) [55]; *Hgf* 10 bp deletion in homozygous mice causes low expression of *Hgf* in the cochlea, which leads to developmental defect of the stria vascularis and reduced endocochlear potential in the cochlea [55]. The 3 bp deletion in the intronic region possibly has a similar mechanism to cause hearing loss. *POU3F4* encodes a POU domain transcription factor expressed in a spiral ligament and spiral limbus [56]. Most DFNX2 (MIM 304400) patients with *POU3F4* variants have profound hearing loss with or without developmental abnormality of the conductive components [57, 58]. Although the temporal bone abnormities could not be confirmed in this family, the PTA results of affected males in the family only presented sensorineural hearing loss, which is less common than mixed hearing loss in patients with POU3F4 variants. The novel c.706G>A (p. Glu236Lys) variant identified in this study is located in the highly conserved POU-specific domain (Figure 4(c)), which is similar to a previously reported c.707A>C; p. (Glu236Ala) variant in a Turkey family [59]. Overall, our results are consistent with known genotype-phenotype correlation of the corresponding genes.

As summarized above, in this study, rare pathogenic variants were identified in four separate deafness-associated genes, *TECTA*, *MYO7A*, *HGF*, and *POU3F4*, which have distinct expression profiles and functions in the inner ear. Nevertheless, all four variants resulted in an almost uniformed type of prelingual, severe-to-profound deafness, representing yet another example of extremely high genetic heterozygosity for hearing loss. Interestingly, within the large pedigree of Family PK-DB-OKA-01 with multiple consanguineous marriages, five of the fifteen affected individuals actually have a hemizygous, X-linked variant as a separate cause of hearing loss irrelevant to the consanguineous marriage pattern (Figure 3(a)). Since most novel deafness-causative genes were originally identified through genetic analysis of such large pedigrees based on assumptions that all affected family members share a single pathogenic variant, our results suggested that caution should remain against such complexed inheritance patterns involving two or multiple genetic causes.

## 5. Conclusions

In summary, our study of three consanguineous families with prelingual, severe-to-profound deafness revealed a rather heterogeneous variant spectrum in the corresponding Pakistani deaf communities. Next-generation sequencing illustrates its advantages in resolving such complexed cases.

## Figures and Tables

**Table 1:** Characterization and classification of the pathogenic variants.

Gene	Variants	MutationTaster	PROVEAN^a^	SIFT^b^	PolyPhen-2	ClinVar	MAF^c^	ACMG classification^d^
*TECTA* (NM_005422.4)	c.536del (p. Leu180Serfs∗20)^e^	Disease causing	—	—	—	—	0	Likely pathogenic
*MYO7A* (NM_001127180.2)	c.3719 G>A (p. Arg1240Gln)	Disease causing	Deleterious (-3.72)	Damaging (0.000)	Probably damaging	Pathogenic	0.0000745	Likely pathogenic
*HGF* (NM_000601.6)	482+1986-1988del	*—*	—	—	—	Pathogenic	0	Likely pathogenic
*POU3F4* (NM_000307.5)	c.706G>A (p. Glu236Lys)^e^	Disease causing	Deleterious (-4.0)	Damaging (0.000)	Probably damaging	—	0	Likely pathogenic

^a^PROVEAN: negative score indicates deleterious, with the cut-off score set as −2.5. ^b^SIFT: deleterious to neutral from scores 0 to 1, with the cut-off score set as 0.05. ^c^The MAF was shown from gnomAD database. ^d^The classification followed the ACMG guidelines for interpretation of sequence variants. ^e^Novel variants identified in this study.

## Data Availability

The original data is available upon request by contacting the corresponding author.

## Supplementary Materials

Supplementary Materials Table S1: 414 genes associated with deafness that were sequenced by targeted NGS.
